# Diagnosis and management of mirror syndrome: a case series with emphasis on the potential role of the sFLT-1/PlGF ratio in clinical practice

**DOI:** 10.61622/rbgo/2026rbgo6

**Published:** 2026-02-20

**Authors:** Gabrielle Soares Behenck, Fernanda Curtois, Patrícia El Beitune, Mirela Foresti Jimenez, Janete Vettorazzi, Marcelo Brandão da Silva, Betania Muller, Lucas Teixeira, Carolina Longo, Gustavo Yano Callado, Edward Araujo Júnior, Talita Micheletti

**Affiliations:** 1 Instituto Materno Fetal Celso Rigo Santa Casa de Porto Alegre Porto Alegre RS Brazil Instituto Materno Fetal Celso Rigo, Santa Casa de Porto Alegre, Porto Alegre, RS, Brazil.; 2 Universidade Federal de Ciências da Saúde de Porto Alegre Department of Obstetrics and Gynecology Porto Alegre RS Brazil Department of Obstetrics and Gynecology, Universidade Federal de Ciências da Saúde de Porto Alegre, Porto Alegre, RS, Brazil.; 3 Lucas Teixeira Clínica Porto Alegre RS Brazil Lucas Teixeira Clínica, Porto Alegre, RS, Brazil.; 4 Universidade Federal de São Paulo Escola Paulista de Medicina Department of Obstetrics São Paulo SP Brazil Department of Obstetrics, Escola Paulista de Medicina, Universidade Federal de São Paulo, São Paulo, SP, Brazil.; 5 Hospital Israelita Albert Einstein Faculdade Israelita de Ciências da Saúde Albert Einstein São Paulo SP Brazil Faculdade Israelita de Ciências da Saúde Albert Einstein, Hospital Israelita Albert Einstein, São Paulo, SP, Brazil.

**Keywords:** Mirror syndrome, sFlt-1/PlGF, Biomarkers, Perinatal outcomes

## Abstract

**Objective:**

To characterize the maternal clinical, ultrasound, and laboratory parameters, maternal and perinatal outcomes of mirror syndrome, and to discuss the role of sFlt-1/PlGF biomarkers in diagnosis and management.

**Methods:**

We conducted a case series including all cases of mirror syndrome diagnosed at a tertiary reference center in Brazil. Clinical, laboratory, ultrasound, and biomarker data were collected, along with maternal and perinatal outcomes.

**Results:**

Nine cases of mirror syndrome were identified, with a mean gestational age at diagnosis of 27+6 weeks. The most frequent maternal findings were lower limb edema (n=7), hypertension (n=8), and proteinuria (n=8). Ultrasound demonstrated fetal hydrops (n=7), polyhydramnios (n=6), and placentomegaly (n=7). Laboratory abnormalities included abnormal proteinuria/creatinuria ratio in 5/8 women tested, elevated 24-hour proteinuria in all 5, anemia in 7, thrombocytopenia in 3, and elevated creatinine in 1. The sFlt-1/PlGF ratio was measured in 5 women; 4 had abnormal results, with 3 above 85, all of whom developed maternal complications or fetal death. Resolution occurred after a mean of 3.4±3.6 days, due to fetal death (n=3), delivery (n=5), or fetal surgery (n=1). In one monochorionic twin pregnancy complicated by twin–anemia–polycythemia sequence, biomarker normalization after intrauterine death of the hydropic twin allowed safe prolongation of the pregnancy and term delivery of the co-twin.

**Conclusion:**

Mirror syndrome should be suspected in pregnancies presenting with preeclampsia-like features in the setting of fetal hydrops or polyhydramnios. The sFlt-1/PlGF ratio may assist in differentiating mirror syndrome from preeclampsia and in identifying women at risk for adverse outcomes.

## Introduction

Mirror syndrome is characterized by a triad of clinical signs including fetal hydrops, placentomegaly, and maternal edema. Several causes of hydrops have been reported, including infection with parvovirus B19, cytomegalovirus, placental chorioangioma, twin-to-twin transfusion syndrome, aneurysm of the vein of Galen, sacrococcygeal teratoma, and fetal leukemia, among others.^(1)^ The incidence is uncertain and variable because it is an underdiagnosed condition.^(2)^ It is associated with a high fetal mortality (between 39-98% in different case series) and tends to resolve once the cause of fetal hydrops is controlled, suggesting a fetal or placental factor at the origin of this condition.^(3,4)^

The etiology and pathogenesis of mirror syndrome are still not well understood.^(5)^ It is thought to share some aspects of the pathophysiology of pre-eclampsia, leading some authors to consider it a "pre-eclampsia-like" syndrome in which there is an imbalance between angiogenic and anti-angiogenic factors.^(6-8)^ An anti-angiogenic state is induced,^(2,9)^ and increased serum levels of soluble fms-like tyrosine kinase-1 (sFlt-1) have been reported in the literature in cases of mirror syndrome associated with parvovirus B19 and cytomegalovirus infection, Rh isoimmunization, and twin-to-twin transfusion syndrome.^(6-8)^ In addition, anatomopathological features of placentas from pregnant women with mirror syndrome similar to those seen in pre-eclampsia (PE) have been observed, with the presence of intervillous edema, intervillous fibrin deposition, and multifocal villous calcifications.^(8,10)^

The presence of villous edema, an increase in fibrin, and an increase in vascular endothelial growth factor receptor-1 (VEGFR-1) have also been observed in placental samples from pregnant women with mirror syndrome, in addition to the identification of extremely high levels of soluble endoglin (sEng) in syncytiotrophoblasts, consistent with the angiogenic profile present in women with severe pre-eclampsia.^(11)^ This suggests that the anti-angiogenic proteins found in maternal serum may be of placental origin. Based on these data, the possibility has been raised that a significant increase in sFlt-1 and the sFlt-1/PlGF (placental growth factor) ratio may be laboratory findings in mirror syndrome.^(10,12)^ However, the cause of this imbalance has not yet been identified, and many authors believe that it is related to placental hypoxia and ischemia caused by placental edema that may promote extrinsic placental venous compression, leading to a reduction in trophoblastic oxygenation.^(11)^ The state of trophoblastic hypoxia leads to the secretion of anti-angiogenic factors that promote a cascade of changes that cause maternal endothelial damage and the consequent clinical manifestations of this condition.^(9)^

The clinical presentation of mirror syndrome also includes symptoms similar to those of PE, such as elevated blood pressure, edema, weight gain and proteinuria.^(2)^ The differences are that mirror syndrome usually presents earlier in pregnancy and it is possible to find dilutional anemia as opposed to the hemoconcentration present in PE.^(2,9)^ Elevated levels of uric acid, lactate dehydrogenase (LDH), creatinine, and D-dimer may be seen, as well as decreased platelet, hemoglobin, hematocrit, and serum albumin levels.^(5)^ As with hypertensive disorders of pregnancy, it is possible that LDH, uric acid, and D-dimer levels may serve as predictors of adverse outcomes in pregnant women at high risk for developing mirror syndrome.^(5)^ Maternal manifestations can be severe, with reports of pulmonary edema, heart failure, and HELLP syndrome.^(5)^

Even though there are some studies analyzing angiogenic and antiangiogenic factors in mirror syndrome, there is very little literature applying it to clinical use. The combination of PlGF and sFlt-1 is already recommended by National Institute for Health and Care Excellence (NICE) guidelines to be used to help guide the diagnosis of pre-eclampsia specially in the third trimester.^(13)^ Considering the clinical and physiopathological similarities, the aim of this study is to discuss the use of biomarkers for the diagnosis and management of mirror syndrome, as well as characterize the maternal clinical, ultrasound and laboratory parameters and maternal and perinatal outcomes of cases treated at a tertiary reference service.

## Methods

This case series was reported in accordance with the CARE guidelines for case reports.^(14)^ It includes all cases of fetal hydrops seen at the Maternal-Fetal Medicine Celso Rigo Institute in Porto Alegre, Brazil, between November 2020 and October 2023. All data were anonymized and stored in a password-protected institutional database accessible only to the research team. Patient confidentiality was strictly maintained throughout data collection and analysis.

Variables collected were sociodemographic (gestational age at diagnosis), clinical (underlying pregnancy condition, maternal symptoms and signs at diagnosis, maternal complications, time until resolution of mirror syndrome, and perinatal outcomes), ultrasonographic (presence of hydrops, polyhydramnios or placentomegaly), laboratory (SFlT-1/PlGF ratio hemoglobin, proteinuria/creatininuria ratio (P/C), 24-h proteinuria ratio, uric acid, hemoglobin, hematocrit, platelet count, lactate dehydrogenase, glutamic-pyruvic transaminase and glutamic-oxaletic transamisase), and anatomopathological (placenta evaluation after delivery). Reference values for laboratory parameters followed institutional standards: hemoglobin 12–16 g/dL, hematocrit 36–46%, platelet count 150–400 ×10^9^/L, uric acid 2.5–6.0 mg/dL, creatinine 0.5–0.9 mg/dL, alanine aminotransferase (ALT) <35 U/L, aspartate aminotransferase (AST) <35 U/L, LDH 100–250 U/L, urine protein/creatinine ratio <0.3, and 24-h proteinuria <300 mg. For the SFlT-1/PIGF, we applied thresholds established for preeclampsia: <38 indicating low risk, 38–84 elevated risk, and ≥85 consistent with preeclampsia or imminent adverse outcomes.

Mirror syndrome was diagnosed by the presence of fetal hydrops, placentomegaly or polyhydramnios associated with maternal edema, hypertension or proteinuria. The ultrasonographic parameters were defined as follows. Fetal hydrops was defined as the presence of fluid accumulation in 2 or more fetal compartments, including ascites, pleural effusion, pericardial effusion, and/or subcutaneous tissue edema. Polyhydramnios was defined as amniotic fluid deepest vertical pocket ≥ 8 cm and/or amniotic fluid index ≥ 25 cm. Placentomegaly was defined as placental thickness ≥ 4 cm.

The laboratory tests were collected by the clinical care team of the service and processed by the hospital's routine clinical laboratory, following standard protocols, including the SFLT-1/PLGF ratio, in cases of clinical suspicion or indication. Since there are no well-established biomarkers cutoff for mirror case syndrome in the literature, we used cutoffs already established for PE and informed at the laboratory report, considering < 38 low risk of PE, 38 to 84 elevated risk of PE and >85 PE or imminent adverse outcome.

The data were computed and analyzes using an Excel 2010 spreadsheet (Microsoft Corp., Redmond, WA, USA) and the results are expressed in number of cases out of total (percentage) and mean ± standard deviation.

This study was approved by the Ethics Committee of UFCSPA) 4.815.034 (CAAE: 46398821.6.0000.5335) and consent was collected from all patients.

## Results

Nine cases of mirror syndrome were identified. The initial suspicion was based on maternal lower limb edema in 7 cases, arterial hypertension in 8, and/or proteinuria in 8. These maternal findings were associated with fetal hydrops in 7 cases, polyhydramnios in 6, and placentomegaly in 7, with a mean gestational age at diagnosis of 27+6 weeks (range, 23+1 to 32+3 weeks). The most relevant laboratory changes at the time of diagnosis included abnormal urine protein/creatinine ratio in 5 of 8 tested women, elevated 24-hour proteinuria in all 5 tested, anemia in 7, thrombocytopenia in 3, elevated uric acid in 5 of 8, elevated transaminases in 2, and elevated creatinine in 1. The underlying fetal diagnoses, gestational age at diagnosis, maternal symptoms and signs, ultrasound findings, and main laboratory results are detailed in chart 1.

**Chart 1:** Maternal-fetal characteristics at diagnosis

Case	Underlying condition	Diagnosis	Gestational age at diagnosis	Maternal signals/symptoms	Ultrasound findings	P/C and/or 24h PTU (g) at diagnosis	sFLT-1/PlGF ratio at diagnosis	Other laboratory parameters at diagnosis
1	Fetal heart anomalies	Supraventricular tachycardia	29w4d	Lower limb edema High arterial pressure	Hydrops Polyhydramnios Placentomegaly	PTU 0.40g	16.3	Hb 10,1g/dl Ht 29.5% Plat 149000/mm^3^ Uric acid 8.4mg/dl
2	Anemia	Non-immune refractory anemia (uncertain etiology)	24w6d	Lower limb edema High arterial pressure	Hydrops Placentomegaly	P/C 0.18 PTU 0.47g	149.0	Hb 8.9g/dl Ht 26.6% Plat 142000/mm^3^ Uric acid 7.0mg/dl
3	Twin pregnancy	TTTS	23w1d	Lower limb edema High arterial pressure	Hydrops Polyhydramnios Placentomegaly	P/C 0.35 PTU 0.33g	Not collected	Hb 8.9g/dl Ht 26.5% Plat 197000/mm^3^ Uric acid 4.9mg/dl
4	Twin pregnancy	TAPS	26w5d	Lower limb edema High arterial pressure Gestational cholestasis	Hydrops Placentomegaly	P/C 6.57 PTU 7.77g	235.4	Hb 10,1g/dl Ht 30.8% Plat 198000/mm^3^ Uric acid 8.0mg/dl
5	Fetal tumor	Sacrococcygeal teratoma	26w6d	Lower limb edema	Polyhydramnios	P/C 0.14	Not collected	Hb 9.7g/dl Ht 29.9% Plat 340000/mm^3^ Uric acid 5.2mg/dl
6	Fetal heart anomalies	Pulmonary atresia	32w1d	Lower limb edema High arterial pressure	Hydrops Polyhydramnios Placentomegaly	P/C 0.95	370.7	Hb 10.5g/dl Ht 28.7% Plat 125000/mm^3^ Uric acid 10mg/dl Creatinine 1.1mg/dl
7	Fetal heart anomalies	Aortic atresia	24w1d	High arterial pressure	Hydrops Placentomegaly	P/C 0.27 PTU 0.3g	Not collected	Hb 12.4g/dl Ht 35.5% Plat 270000/mm^3^ GOT 93U/l, GPT 249U/l
8	Fetal heart anomalies	Isomerism, atrioventricular septal defect, pulmonary stenosis, atrioventricular block	32w3d	Lower limb edema High arterial pressure	No hydrops criteria (only subcutaneous edema) Polyhydramnios	P/C 0.66	Not collected	Hb 12.5g/dl Ht 35.5% Plat 350000/mm^3^ Uric acid 7.6mg/dl
9	Fetal heart anomalies	Aortic stenosis	30w4d	High arterial pressure	Hydrops Polyhydramnios Placentomegaly	P/C 1.2	47	Hb 9.3g/dl Ht 27.8% Plat 178000/mm^3^ GPT 72U/l Uric acid 3.9mg/dl

P/C: proteinuria/creatininuria ratio (normal < 0.3); 24h PTU: 24h urinary protein (normal < 300mg/24h); Hb: hemoglobin (normal 12-16 g/dL); Ht: hematocrit (normal 34-47%); Plat: platelets (normal 150.000-450.000/m³); UA: uric acid (normal 2.4-5.7 mg/dL); LDH: lactate dehydrogenase (normal 100-190 U/L); sFLT-1/PlGF ratio: soluble fms-like tyrosine kinase-1/placental growth factor (< 38 low risk of preeclampsia; 38-84 elevated risk of preeclampsia; > 85 preeclampsia or imminent adverse outcome); GPT: glutamic pyruvic transaminase (normal < 33 U/L); GOT: glutamic-oxalacetic transaminase (normal < 40 U/L). TAPS: twin-twin polycythemia sequence. TTTS: twin-twin transfusion syndrome

sFlt-1/PlGF biomarkers were analyzed in 5 women prior to resolution. One had a normal result, while 4 had abnormal values. Of these, 3 showed ratios greater than 85, consistent with preeclampsia or imminent adverse outcome. All women with a ratio greater than 85 experienced unfavorable outcomes, including 2 maternal complications (nephrotic syndrome and acute renal insufficiency) and 2 fetal deaths. In contrast, the woman with a mildly elevated ratio and the one with normal results had no maternal complications or fetal deaths. Maternal–fetal outcomes are presented in table 2.

**Chart 2:** Maternal-fetal outcomes

Outcome	Number of cases/total cases (%) or mean ± standard deviation	Comments
Presence of severe maternal complication	3/9 (33%)	Case 4: nephrotic syndrome Case 5: pleural effusion Case 6: acute renal insufficiency
Time interval from diagnosis to resolution (days)	3.4 ± 3.6	Resolution by delivery (n=7) or fetal death (1) or fetal surgery (n=1, twin pregnancy with TAPS)
Neonatal outcomes		
At least 1 fetus alive	1/9 (11%)	Case 4: twin pregnancy with TAPS
Fetal death	1/9 (11%)	Case 2: death due to underlying fetal condition
Neonatal death	7/9 (78%)	Due to prematurity or underlying fetal condition Case 3: no time for mirror syndrome improvement confirmation due to preterm labor following fetal surgery

TAPS: twin-anemia-polycythemia sequence

**Figure 1:**
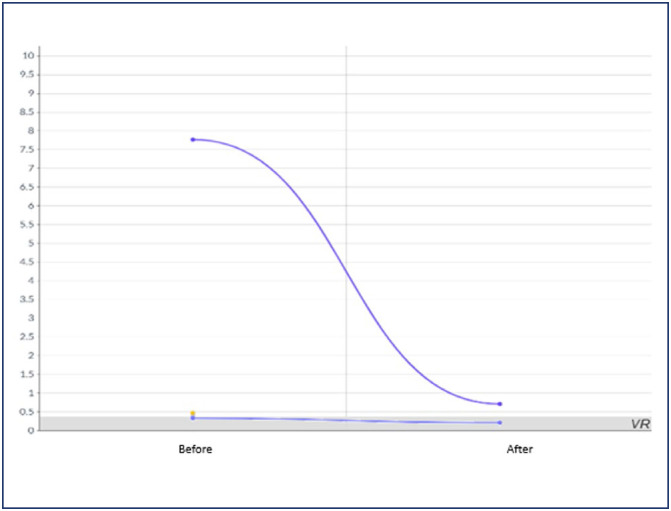
Behavior of 24h urinary protein (g) before and after resolution of mirror syndrome

**Figure 2:**
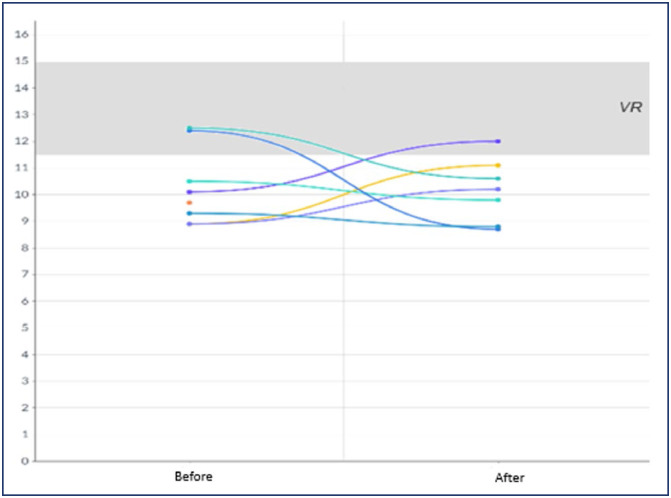
Behavior of hemoglobin (g/dl) before and after resolution of mirror syndrome

**Figure 3:**
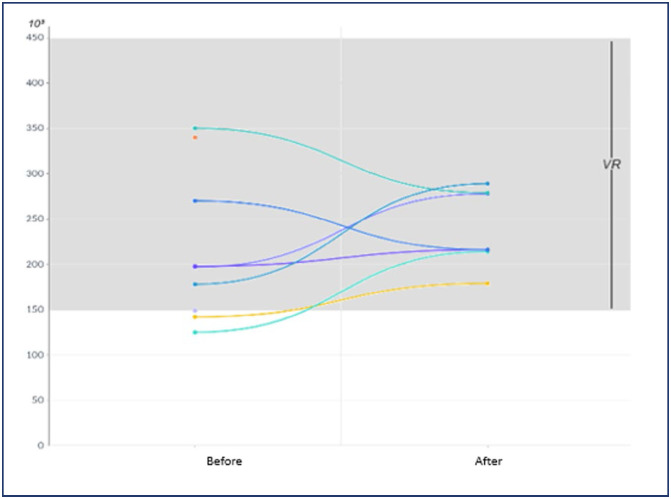
Behavior of platelets (mm^3^) before and after resolution of mirror syndrome

**Figure 4:**
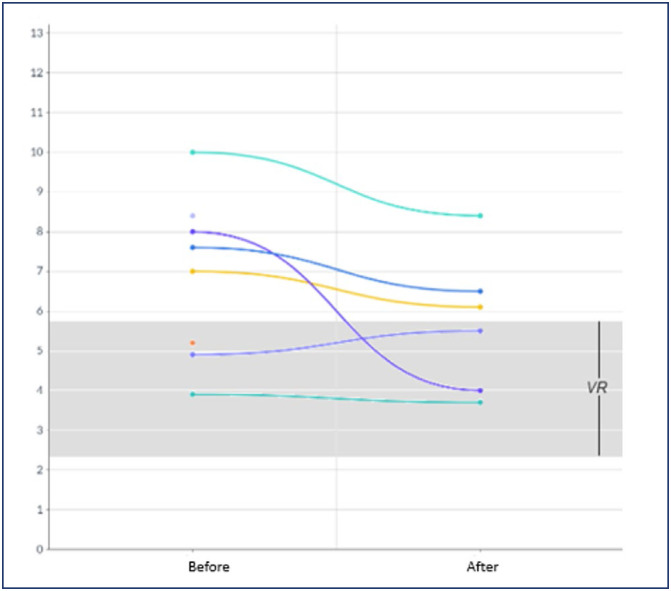
Behavior of uric acid (mg/dl) before and after resolution of mirror syndrome

All women showed clinical improvement after resolution of mirror syndrome, with laboratory parameters before and after resolution illustrated in figures 1, 2, 3 and 4. In 2 women, 24-hour proteinuria was collected after resolution, showing reduction or normalization.

In the only case in which the sFlt-1/PlGF ratio was evaluated both before and after resolution (case 4) chart 1 – monochorionic twin pregnancy with twin–anemia–polycythemia sequence, complicated by maternal nephrotic syndrome and intrauterine death of the hydropic fetus despite transfusion), the ratio decreased from 235.4 to 22 after the death of one twin. In this case, proteinuria also decreased, although 24-hour proteinuria did not normalize immediately. Placental histopathology was available in 6 cases, showing villous fibrosis and intervillous fibrin deposits in 3, without additional specific findings.

## Discussion

This case series highlights the clinical challenges associated with diagnosing and managing mirror syndrome, a rare condition characterized by maternal symptoms that mirror fetal hydrops.^(13)^ Our findings suggest that a careful combination of clinical assessment, ultrasound findings, laboratory parameters, and the use of biomarkers such as the sFlt-1/PlGF ratio can be valuable in identifying and managing this condition.

The initial clinical suspicion in our cohort was primarily based on edema and hypertension, frequently accompanied by fetal complications. Anemia was another important observation, with reductions in hematocrit and hemoglobin levels suggesting hemodilution. Although anemia has been described in mirror syndrome,^(15)^ the phenomenon of hemodilution remains underexplored in many studies,^(16)^ despite being considered a distinguishing feature and appearing earlier in gestation compared with PE.^(2)^ Mirror syndrome is frequently mistaken for PE, but the two conditions differ: mirror syndrome typically presents with hemodilutional anemia, whereas PE involves hemoconcentration.^(10)^ Hirata et al.^(17)^ confirmed this distinction, showing significantly lower hemoglobin and albumin in mirror syndrome. Our data support the hypothesis that an imbalance between angiogenic and antiangiogenic factors may also play a role in mirror syndrome, similar to PE.^(18)^

Severe maternal complications such as pulmonary and renal dysfunction, described in the literature,^(14)^ were also observed in our cohort. Importantly, all cases with serious maternal complications had markedly elevated sFlt-1/PlGF ratios (>85), underscoring its potential as a predictive marker for adverse outcomes, similar to its established role in PE.^(9,19)^ Unlike PE, however, normalization of biomarker levels and maternal improvement may occur after treatment of the underlying fetal condition, including fetal therapy or delivery. Gavin et al.^(20)^ demonstrated that fetal therapy may serve as a primary treatment option, though responses can be variable, and some patients improve only days after resolution of hydrops.^(15)^ Sichitiu et al.^(15)^ reported up to 10 days between intervention and resolution, emphasizing the need for close monitoring.

Two cases in our series involved twin gestations. These require separate consideration because of the unique hemodynamic and biochemical profile of multiple pregnancies. Angiogenic and antiangiogenic factor levels are known to differ between singletons and twins,^(21)^ and multiple gestations carry a higher baseline risk of preeclampsia. Although robust data are lacking, most authors agree that biomarker cutoffs validated in singleton pregnancies can reasonably be applied to twins.^(22)^ In one monochorionic twin pregnancy complicated by twin–anemia–polycythemia sequence, normalization of the sFlt-1/PlGF ratio after the intrauterine death of the hydropic twin allowed the clinical team to distinguish mirror syndrome from PE. This distinction guided management, permitting prolongation of the pregnancy and term delivery of the co-twin in good condition.

The use of biomarkers such as the sFlt-1/PlGF ratio therefore adds an important layer to clinical decision-making. Ratios above 85 were associated with adverse maternal and fetal outcomes in this series, including nephrotic syndrome, renal failure, and fetal death. Conversely, normal or only mildly elevated ratios were associated with better outcomes, suggesting potential value for risk stratification.

The timing of intervention remains critical. Resolution of mirror syndrome—whether by fetal therapy, delivery, or fetal demise—was consistently followed by maternal improvement, emphasizing the importance of early recognition. However, the poor fetal prognosis, reflected in several cases of intrauterine death, remains a major challenge.

From a clinical perspective, hematological changes such as dilutional anemia and thrombocytopenia were consistent with previous reports.^(2)^ Elevated uric acid, proteinuria, and renal dysfunction were also observed, reinforcing the overlap with PE and their possible role in monitoring disease progression.

This study contributes to the growing recognition that mirror syndrome, although rare and underdiagnosed, requires a high index of suspicion in cases of fetal hydrops. Angiogenic biomarkers may aid in differentiating mirror syndrome from PE and identifying women at risk of severe complications.^(23)^ However, larger studies are needed to validate the clinical utility of the sFlt-1/PlGF ratio and to develop standardized management strategies.

The main limitation of this study is its retrospective case series design, which limits generalizability. The inclusion of twin pregnancies is another potential confounder, as these cases differ substantially from singletons in pathophysiology and baseline risk of PE. Additionally, not all women underwent the same ancillary tests, such as placental pathology or serial biomarker assessment, reducing the completeness of comparisons. Cost and delayed availability of biomarker results further limited their applicability in real-time clinical decision-making. Another important limitation of this study is the heterogeneity of the underlying conditions that caused fetal hydrops, including congenital heart disease, non-immune anemia, sacrococcygeal teratoma, and complications of monochorionic twin pregnancies such as twin–twin transfusion syndrome and twin–anemia–polycythemia sequence. The fact that hydrops can result from different etiologies, predominantly fetal, may account for the poor fetal prognosis. However, these underlying causes would not be expected to influence the maternal clinical presentation in a way that would warrant alternative diagnoses other than mirror syndrome.

## Conclusion

In conclusion, while mirror syndrome remains a challenging condition with high perinatal mortality, the use of biomarkers such as sFlt-1/PlGF offers a potentially valuable tool for early diagnosis and management. Combined with clinical, ultrasound and laboratory assessments, this could enhance the identification of at-risk pregnancies and improve maternal and fetal outcomes.

## Data Availability

the authors did not make the data from this article available in repositories prior to submission.
